# Changes in Gab2 phosphorylation and interaction partners in response to interleukin (IL)-2 stimulation in T-lymphocytes

**DOI:** 10.1038/srep23530

**Published:** 2016-03-30

**Authors:** Nerea Osinalde, Virginia Sánchez-Quiles, Blagoy Blagoev, Irina Kratchmarova

**Affiliations:** 1 Department of Biochemistry and Molecular Biology, University of Southern Denmark, 5230 Odense M, Denmark

## Abstract

Interleukin-2 (IL-2) stimulation results in T-cell growth as a consequence of activation of highly sophisticated and fine-tuned signaling pathways. Despite lacking intrinsic enzymatic activity, scaffold proteins such as Gab2, play a pivotal role in IL-2-triggered signal transduction integrating, diversifying and amplifying the signal by serving as a platform for the assembly of effectors proteins. Traditionally, Gab2-mediated protein recruitment was believed to solely depend on cytokine-induced phosphotyrosine moieties. At present, phosphorylation on serine/threonine residues is also emerging as a key mediator of Gab2-dependent signal regulation. Despite its relevance, IL-2-triggered regulation on Gab2 phosphorylation is yet poorly understood. Combining antibody- and TiO_2_-based enrichment of the scaffold protein with SILAC quantitative mass spectrometry we disclose the prominent regulation IL-2 exerts on Gab2 serine/threonine phosphorylation by showing that at least 18 serines and 1 threonine, including previously non-reported ones, become phosphorylated in response to cytokine stimulation. Additionally, we decipher the interactome of the docking protein in resting and cytokine-treated T-lymphocytes and besides well-known Gab2 interactors we discover three novel cytokine-inducible Gab2-binding proteins. Thus, our data provide novel insights and a wealth of candidates for future studies that will shed light into the role of Gab2 in IL-2-initiated signal transduction.

Interleukin 2 (IL-2) is a pleiotropic cytokine that promotes differentiation of naive CD4^+^ and CD8^+^ T-lymphocytes into effector and memory T-cells respectively, augments the cytolytic activity of CD8^+^ T-cells and natural killer cells (NK-cells), triggers activation-induced cell death (AICD) and stimulates proliferation of different immune cell types^1^,^2^,^3^,^4^. Indeed, due to its capacity to promote T-cell growth, IL-2 has clinically emerged as the most widely used cytokine in adoptive immunotherapy for cancer^5^,^6^. Despite its effectiveness, IL-2 treatment is often accompanied by undesired side effects that must be overcome in order to ensure safer therapies. Hence, the characterization of the molecular events underlying IL-2-dependent responses constitutes a key way into the development of more accurate treatments.

Binding of IL-2 to its transmembrane receptor (IL-2R) results in the rapid activation of highly interconnected and carefully orchestrated signaling pathways that ultimately converge into the nucleus eventually elaborating the desired cellular output^7^,^8^. The initial step in IL-2 signaling involves activation of JAK tyrosine kinases which phosphorylate the receptor, thus creating anchoring sites for signal relay molecules bearing phosphotyrosine-binding (PTB) and Src-homology 2 (SH2) domains that are subsequently phosphorylated as well^9^,^10^. Hence, the binding of the cytokine to the cell surface promotes the formation of large intracellular molecular complexes that allow a fast and amplified propagation of the signal. Among the recruited proteins, several of them constitute enzymes or transcription factors whose activity is indeed regulated by site-specific modifications^11^,^12^. Additionally, some of the molecular characters associated to the activated receptors represent scaffold or docking proteins with no inherent enzymatic or transcriptional activity, but importantly serving as physical platforms facilitating the assembly of the signalling hubs. Scaffold molecules thereby enable the localization of other effector proteins at specific cellular compartments, protect them from inactivation and integrate positive and negative feedback mechanisms, ultimately allowing the modulation of the signal transduction^13^.

Among the scaffold proteins involved in the signaling transduction triggered by IL-2 stands the human Grb2-associate binder (Gab) family, which is comprised by three members (Gab1–3). Upon stimulation Gab proteins become phosphorylated and act as molecular bridges between growth factor- or cytokine-activated receptor complexes and downstream signaling molecules^14^,^15^,^16^. In fact, among others we have demonstrated that docking proteins Gab2 and Gab3 become tyrosine phosphorylated and hence, participate in the signaling networks initiated by IL-2 in CD4^+^ T-lymphocytes^17^,^18^,^19^,^20^,^21^. Upon stimulation, phosphotyrosine moieties mediate the interaction with diverse signaling molecules, such as, PTPN11, p85 regulatory subunit of PI-3 kinase, PLCγ, Shc and CrkL which leads to the activation of RAS/MAPK and PI3K/AKT signaling pathways^22^. Indeed, Gab2 overexpression has been shown to promote the alteration of these signaling branches, importantly contributing to the development of distinct cancers including breast and ovarian cancer, leukemia and melanoma^23^,^24^,^25^.

At present, serine and threonine phosphorylations of Gabs are emerging as key regulatory mechanism that can determine the fate of the signal. Lynch and co-workers first demonstrated that AKT-mediated phosphorylation on Gab2 S159 contributes to the attenuation of heregulin (HRG)-activated signaling pathways by uncoupling Gab2 from ErbB receptors^26^. Similarly, growth factor-triggered phosphorylation on Gab2 S210 and T391 has been shown to mediate negative feedback regulation by recruiting 14–3–3 proteins that disrupt Gab2/Grb2 interaction^27^. The biological significance of such finding became clear with the recent discovery that Gab1 T387, corresponding to Gab2 T391, is mutated (T387N) in breast cancer which results in EGF-independent proliferation of mammary epithelial cells as a consequence of impaired signal attenuation mechanism^28^. Consequently, the biological relevance of Gab2 phosphorylation occurring not only on tyrosines but also on serine and threonine residues is indisputable.

Biochemical analyses have already suggested that Gab2 is highly phosphorylated on serine and threonine residues in response to IL-2^17^. However, the precise effect that IL-2 exerts on the distinct site-specific phosphorylable residues on the scaffold proteins remains elusive. Here, we present the largest phosphoproteomic study depicting Gab2 phosphorylations in resting and IL-2-treated CD4^+^ T-lymphocytes. We identify 29 distinct phosphorylations on human Gab2 including 3 novel phosphorylated serine residues (pS146, pS147 and pS459) that have never been reported before. More importantly, we provide robust quantification data regarding 24 phosphorylation sites and provide evidence indicating that IL-2 triggers at least the phosphorylation of 18 serines and 1 threonine. Additionally, considering that the role of Gab2 consists on serving as a molecular platform to recruit distinct signaling proteins, we dissected the Gab2 interactome in resting and cytokine-treated T-lymphocytes by taking advantage of Stable Isotope Labeling by Amino acids in Cell culture (SILAC) quantitative mass spectrometry (Q-MS)^29^,^30^,^31^,^32^. Besides detecting already reported cytokine-dependent and – independent Gab2 interactors, we discovered 3 previously unknown cytokine-inducible Gab2-binding proteins which were further validated by Western blotting. Collectively, the data provided here considerably expand the current knowledge of the IL-2-dependent regulation of Gab2 which may originate a plethora of future investigations shedding new light into the role of the scaffold proteins in the signaling networks initiated by IL-2.

## Results

### Kit225 T-lymphocytes express Gab2 and Gab3 but not Gab1

**Figure 1:**
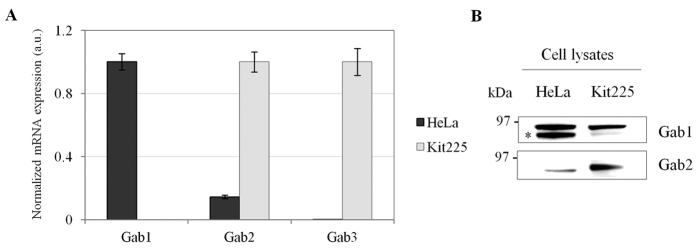
Expression of Gab family members. (**A**) Gab1-3 gene expression was determined in HeLa and Kit225 cells using quantitative RT-PCR. (**B**) Gab1-2 protein levels were detected in HeLa and Kit225 T-cells by Western blot.

Our previous studies deciphering the tyrosine-phosphoproteome of IL-2 pathways demonstrated that Gab2 and Gab3 participate in the signal transduction initiated by the cytokine in Kit225 T-lymphocytes^20^,^21^. Gab1, in turn, has never been identified in those experiments, which does not necessarily imply that is not expressed in Kit225 T-cells. For that reason, in order to determine which Gab family members are expressed in this CD4^+^ T-cell line, mRNA levels of each gene-product were measured by real time quantitative PCR. As shown in Fig. 1A, mRNA levels of Gab2 and Gab3 were high in Kit225 T-cells, whereas Gab1 gene expression was nearly undetectable. On the contrary, HeLa cells expressed high levels of Gab1 but low or almost undetectable amounts of Gab2 and Gab3, which is in agreement with recently published data^33^. In line with that, Western blot analyses revealed that expression of Gab1 and Gab2 is restricted to HeLa and Kit225 T-cell, respectively (Fig. 1B). Antibody-based detection of Gab3 was not successful but our previous mass spectrometry-based works have repeatedly demonstrated that Gab3 is also expressed in Kit225 T-lymphocytes^20^,^21^. Altogether, our data indicate that Gab2 and Gab3 are the only Gab family members predominantly expressed in Kit225 T-cells and hence, participating in IL-2 signal transduction.

### SILAC-based quantitative phosphoproteomics/proteomics approach to characterize IL-2-triggered regulation of Gab2

Gab2 and Gab3 family members share moderate sequence homology (33.4% identity) but they conserve the same modular structure^16^ (Supplementary Fig. S1). Therefore, it seems plausible that IL-2-triggered regulation of both scaffold proteins does not differ so much. Nonetheless, Gab2 is larger than Gab3 and contains more protein binding regions which may explain why Gab2-deficient mice show more defects that Gab3-deficient ones^34^,^35^,^36^. For that reason, we decided to focus our investigation on studying in detail IL-2-mediated modulation of Gab2 as the representative Gab family member expressed in Kit225 T-cells.

**Figure 2:**
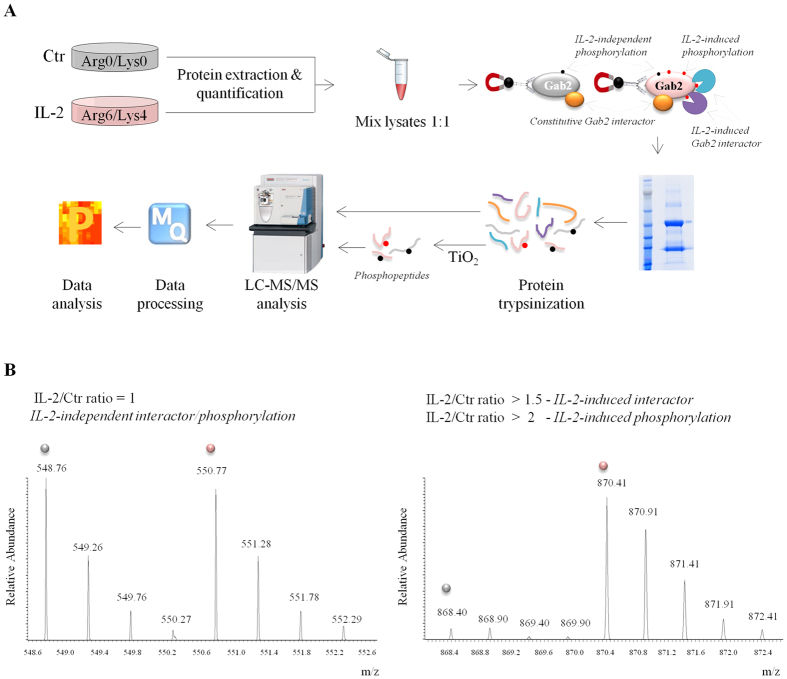
Experimental workflow followed to dissect IL-2-triggered regulation on Gab2. (**A**) A schematic flow diagram depicting the steps for identification and quantification of Gab2 phosphopeptides as well as Gab2-interacting proteins. (**B**) Examples of SILAC MS spectra illustrating peptides that display different ratios. The left peptide shows no regulation by IL-2 (IL-2/Ctr ratio = 1) whereas the right peptide shows upregulation upon IL-2 stimulation (IL-2/Ctr ratio > 1.5 for interacting proteins and IL-2/Ctr ratio > 2 for phosphopeptides). Peptides derived from resting T-cells are marked in grey whereas the ones derived from IL-2-treated condition are marked in pink.

To depict Gab2 site-specific phosphorylation events and unveil the cytokine-dependent and –independent interactors of the scaffold protein we followed the experimental design outlined in Fig. 2A. Two populations of Kit225 T-cells were cultured in “light” or “heavy” media and were kept unstimulated (Ctr) or were stimulated for 5 minutes with IL-2, respectively. After cell lysis, samples were equally mixed, subjected to immunoprecipitation using an antibody against Gab2 and eluted immune complexes were fractionated by SDS-PAGE followed by in-gel digestion. Part of the sample was desalted and directly analyzed by LC-MS/MS aiming to detect Gab2-interacting partners whereas the remaining part was enriched in phosphopeptides using TiO_2_ beads prior to MS analysis. In addition to detect site-specific phosphorylations and putative Gab2 interactors, the use of SILAC allows discerning between IL-2-dependent and -independent phosphorylation events as well as constitutive and IL-2-inducible Gab2 interactors. A phosphopeptide bearing a site-specific phosphorylation that is not affected by IL-2 stimulation will be similarly enriched in resting and cytokine-treated cells, thus it will display a SILAC IL-2/Ctr ratio of 1 (Fig. 2B, left MS spectra). Likewise, peptides derived from a protein that is constitutively associated with the scaffold protein will also show a SILAC ratio of 1. On the contrary, if IL-2 induces phosphorylation on a specific residue or promotes an interaction between Gab2 and a signaling protein, the corresponding phosphopeptide and peptides respectively, will be more abundant in the cytokine-treated condition (Fig. 2B, right MS spectra).

### Depicting Gab2 phosphorylation in resting and IL-2-treated T-lymphocytes

Following the approach described above, we confidently identified 29 amino acids on Gab2 that are phosphorylated *in vivo* including 22 phosphorylated serines (pS), 5 phosphorylated threonines (pT) and 2 phosphorylated tyrosines (pY). Interestingly, 6 of these Gab2 pS (pS133, pS148, pS149, pS223, pS281, pS422) are conserved in the Gab family member Gab3 (corresponding to pS132, pS141, pS142, pS209, pS242, pS362, respectively) that is also expressed in Kit225 T-lymphocytes (Table 1). In addition, the phosphorylated Gab2 residues S141, T319 and T391 align with distinct but still phosphorylable Gab3 residues (T134, S273 and S344, respectively). In fact, we have already demonstrated that Gab3 S344 and S362 are phosphorylated in IL-2-treated CD4^+^ T-lymphocytes^20^. Altogether, it seems plausible that the putative and confirmed Gab3 phosphosites mentioned above are subjected to similar regulation as Gab2 in Kit225 T-cells upon cytokine treatment.

**Figure 3:**
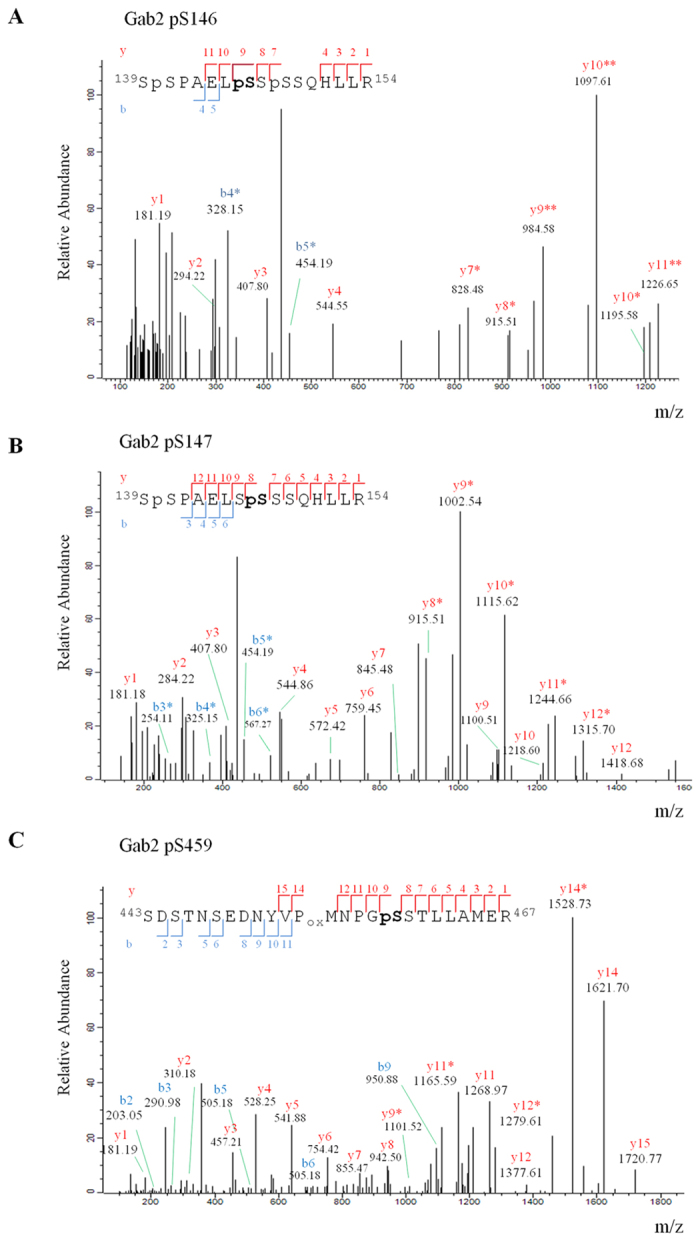
MS/MS spectra of novel Gab2 phosphopeptides identified. Annotated MS/MS spectrum of a (**A**) triply phosphoryated Gab2 peptide (SpSPAEL**pS**SpSSQHLLR) containing pS146, (**B**) doubly phosporylated Gab2 peptide (SpSPAELS**pS**SSSQHLLR) containing pS147 and (**C**) singly phosphorylated Gab2 peptide (SDSTNSEDNYVP_ox_MNPG**pS**STLLAMER) bearing pS459. The b ions (in blue) and y ions (in red) identified are indicated both in the spectrum and in the peptide sequence * or ** denotes loss of one or two H_3_PO_4_ (Δm 97.9768) respectively, from the phosphorylated ion.

It is worth highlighting that this is the first study demonstrating that Gab2 S147 and S459 can be phosphorylated in human CD4^+^ T-lymphocytes. Moreover, this study provides unprecedented evidence indicating that human Gab2 S146 can also be phosphorylated, a residue that was already reported to be modified in mice^37^. The precise assignment of those phosphorylation sites was verified by manual annotation of their corresponding fragmentation spectra. As shown in Fig. 3A, the presence of the 984.58^+1^ ion (y9** in SpSPAELpS^146^SpSSQHLLR) and the absence of the 1093.4534^+1^ ion (y8 in SpSPAELSpS^147^pSSQHLLR) supports the unambiguous identification of the phosphorylation of S146 residue in Gab2. Similarly, the presence of the 915.51^+1^ and 846.488^+1^ ions (y8* and y7 in SpSPAELSpS^147^SSQHLLR) and the absence of the ion 915.51^+1^ (y7 in SpSPAELSSpS^148^SQHLLR or SpSPAELSSSpS^149^QHLLR) allows to assign the position of the phosphosite to S147 of Gab2 (Fig. 3B). Regarding the Gab2 phosphopeptide comprising amino acid residues 443–467, the discriminating ions y8 and y9* indicate that among the six phosphorylable residues within the Gab2 phosphopeptide 443–467, only S459 is phosphorylated (Fig. 3C).

**Figure 4:**
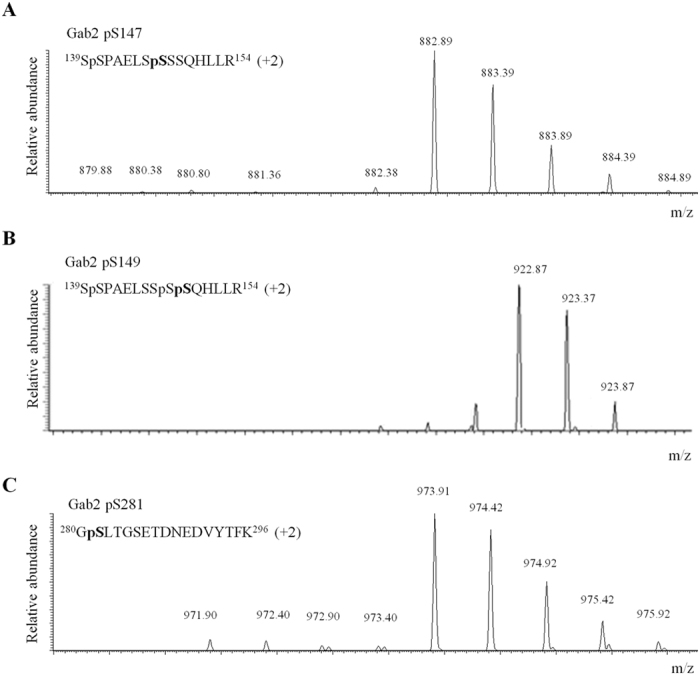
MS spectra of IL-2-induced Gab2 phosphopeptides not displaying a SILAC ratio. MS spectra of (**A**) pS147-, (**B**) pS149- and (**C**) pS281-bearing phosphopeptides displaying no SILAC ratio. Whereas the light version of the phosphopeptide corresponding to resting T-cells (grey dot) is barely detectable, the heavy version of the same peptide (pink dot) is clearly distinguished indicating that upon cytokine stimulation Gab2 S147, S149 and S281 become phosphorylated.

Gab2 is known to be tyrosine phosphorylated in response to IL-2 and accordingly we found that Y476 and Y643 become phosphorylated in cytokine-treated CD4^+^ T-lymphocytes (Supplementary Fig. S2). More interestingly, the study presented here identified a large number of Gab2 pS/pT residues that are differentially regulated in response to IL-2 stimulation (Table 1). Our SILAC-based quantitative phosphoproteomic screen shows that phosphorylations on Gab2 S210, T287, T385 and T391 are not affected (0.5 < IL-2/Ctr ratio < 2, in both replicas) by cytokine stimulation. On the contrary, we provide evidence indicating that 18 serines and 1 threonine (T319) distributed along the central 400 amino acid-region of Gab2 (aa 141–543) become phosphorylated *in vivo* in CD4^+^ T-lymphocytes treated with IL-2. It should be noted that Gab2 pS147, pS149 and pS281 do not display a SILAC ratio in both replicas but still according to peak intensity data collected, the three phosphosites are induced in response to IL-2 treatment. As shown in Fig. 4, the three phosphosites are barely detectable in the light-labeled peak of the SILAC pair corresponding to resting T-cells (no intensity value). On the contrary, all of them were detected in the heavy-labeled SILAC condition corresponding to cytokine-treated T-lymphocytes and thus display a signal intensity which indicates that the three residues are phosphorylated upon IL-2 stimulation (Fig. 4A–C).

Overall, results presented here represent the largest dataset of IL-2-dependent and-independent Gab2 phosphorylations reported to date. Hence, our study adds to a growing body of literature that points towards phosphorylations occurring on serine and threonine residues as key players in the signaling pathways modulated by Gab2. Future investigation will be necessary to clarify the role of each site-specific phosphorylation in the signal transduction initiated by IL-2.

### Dissecting the interactome of Gab2 in IL-2-treated T-lymphocytes

Phosphorylations on Gab2 serve as docking sites for downstream molecules that participate in the distinct signaling branches activated upon engagement of IL-2 and IL-2 receptor. In order to identify the proteins that are recruited to Gab2 in response to IL-2 stimulation, we combined immunoprecipitation of the endogenous scaffold protein with SILAC-based quantitative mass spectrometry as illustrated in Fig. 2A.

**Figure 5:**
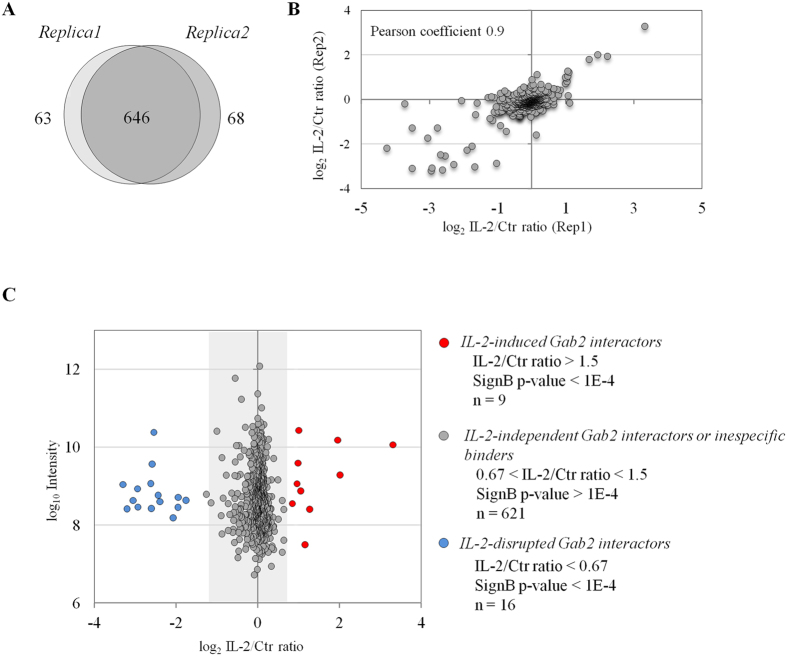
Interactome of Gab2 in Kit225 T-cells. (**A**) Venn diagram showing the overlap in proteins co-immunoprecipitating with Gab2 in the two biological replicates performed. (**B**) Correlation between IL-2/Ctr SILAC ratios obtained in the two replicates analyzed. (**C**) Combined IL-2/Ctr SILAC ratio corresponding to all the proteins quantified in the two biological replicas of each Gab2 immunoprecipitation is represented as a function of protein intensity in the MS. Dots coloured in red represent proteins that are consistently enriched (IL-2/Ctr > 1.5; Significance B p-value < 0.0001). (**D**) Heat map and hierarchical clustering dendrogram indicating the IL-2/Ctr SILAC ratio measured in each replicate of the proteins associating with Gab2 upon IL-2 stimulation.

We quantified 709 and 714 proteins in the first and second replica performed respectively, of which 646 were commonly quantified in the two experiments (Fig. 5A & Supplementary Table S2). Comparison of the SILAC ratios obtained for those proteins showed strong correlation between the two biological replicas with Pearson coefficient of 0.9 (Fig. 5B). The majority of the proteins were found equally enriched in resting and IL-2-treated T-lymphocytes. These dataset embrace constitutive Gab2-binding proteins as well as unspecific interactors (Fig. 5C). It should be noted that among IL-2-independent Gab2-interacting proteins we detected the adaptor protein Grb2 (Supplementary Table S2). The constitutive association between the two proteins was further validated by Gab2 immunoprecipitation followed by Western blotting (Supplementary Fig. S3). These results indicate that the scaffold protein Gab2 and the adaptor protein Grb2 form a stable complex in CD4^+^ T-lymphocytes, which is line with previously published data^38^.

**Figure 6:**
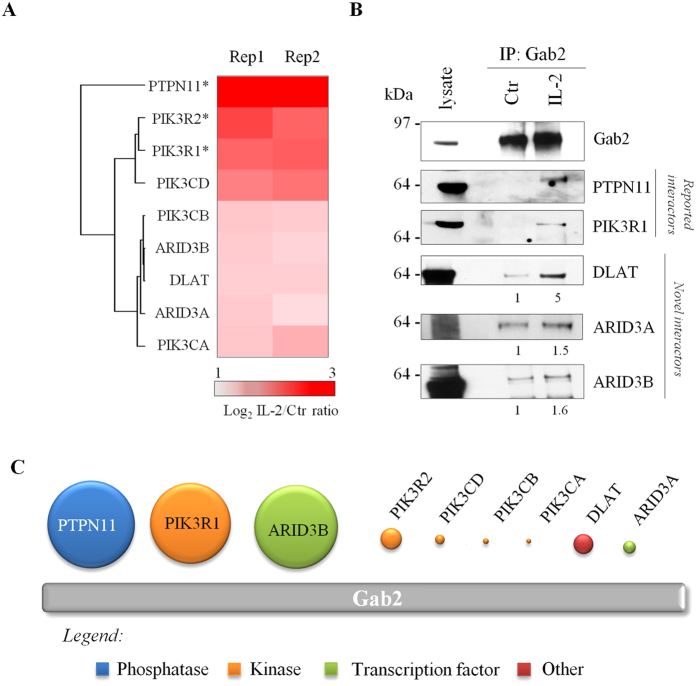
IL-2-inducible Gab2-interacting proteins. (**A**) Heat map and hierarchical clustering dendrogram indicating the SILAC IL-2/Ctr ratio displayed by the cytokine-inducible Gab2-binding protein in each replica. (**B**) Validation of selected well-known and novel Gab2-binding proteins by immunoblotting. (**C**) Relative binding abundance of the detected Gab2 interactors. *indicates described Gab2 interactors.

More interestingly, our interactome analysis revealed 9 proteins that were significantly enriched in Gab2-containing immune complexes upon cytokine stimulation (IL-2/Ctr >1.5; Significance B p-value < 0.0001) indicating that their association with Gab2 depends on IL-2 treatment (Fig. 5C & Supplementary Table S2). The 9 inducible Gab2-interacting proteins detected displayed consistent SILAC ratio in the two biological replicas performed (Fig. 6A). Tyrosine phosphatase PTPN11 and p85 PI3-kinase regulatory subunits alpha and beta (PIK3R1 and PIK3R2, respectively) were the IL-2-inducible Gab2 interactors showing the highest SILAC ratio. We validated these results by Western blot analyses which agree with previous studies showing that PTPN11 and p85 PI3-kinase interact with the tyrosine phosphorylated version of Gab2^39^,^40^,^41^. Additionally, all catalytic subunits comprising the Class I_A_ of PI3K family were also found to be enriched in IL-2 treated conditions (PIK3CA, PIK3CB and PIK3CD). More interestingly, for the first time this study provides evidence that DLAT (a subunit of the pyruvate dehydrogenase complex) and transcription factors ARID3A and ARID3B associate with Gab2 in response to IL-2 stimulation. We could further confirm by immunoblotting that the three novel Gab2 interactors discovered in this study, named DLAT, ARID3A and ARID3B, indeed interact with Gab2 scaffold protein in IL-2-stimulated Kit225 T-lymphocytes (Fig. 6B).

The biological information extracted from the SILAC ratios obtained in the Gab2 immunoprecipitation experiments is unquestionable as they allow recognizing the specific cytokine-dependent interactors of the scaffold protein from a dataset composed by hundreds of proteins. Nonetheless, protein SILAC ratios do not provide information on the relative abundance of the proteins within the complex. This however can be extracted from the peptide intensity information in the dataset since it is assumed that the signal response of the three most abundant peptides can provide good approximation of the amount of protein^42^. Accordingly, we calculated the relative abundance of each IL-2-dependent Gab2 interactor detected in this study (Fig. 6C). Our data indicate that the most prominent proteins associating with Gab2 are PTPN11 and PIK3R1, indeed the two well-documented interactors of the scaffold protein. Strikingly, the binding level of ARID3B, the newly identified interactor of Gab2, was also found to be among the highest. The catalytic subunits of PI3-kinase family were detected lower in abundance as well as the novel interactors ARID3A and DLAT.

## Discussion

IL-2-triggered signaling pathways are strictly fine-tuned by tyrosine phosphorylation events that trigger and modulate a wide range of processes such as protein-protein interaction, protein localization and enzymatic activity of distinct signaling molecules^18^,^19^,^20^,^43^. Initial events following cytokine and receptor engagement involve activation of JAK tyrosine kinases which in turn phosphorylate the receptor creating docking sites for signaling molecules that participate in transducing the signal initiated on the cell surface inside the cell. In response to cytokine stimulation, among others, Gab scaffold proteins are recruited to the activated receptor-complex where they become tyrosine phosphorylated and recruit additional effector molecules thereby integrating, amplifying and diversifying the signal transduction pathways^44^,^45^.

The initially discovered Gab family member Gab1 is believed to be ubiquitously expressed^15^,^46^; however, here we demonstrate that Gab1 mRNA and protein levels are barely detectable in Kit225 T-cells. On the contrary, Gab2 and Gab3 are highly expressed in Kit225 CD4^+^ T-lymphocytes (Fig. 1). Indeed, we had previously demonstrated that Gab2 and Gab3 are tyrosine phosphorylated in response to IL-2 stimulation^20^,^21^. Altogether, our studies provide evidence indicating that the latter two Gab family members are the only ones participating in the signal transduction initiated by IL-2 in Kit225 T-lymphocytes.

**Figure 7:**
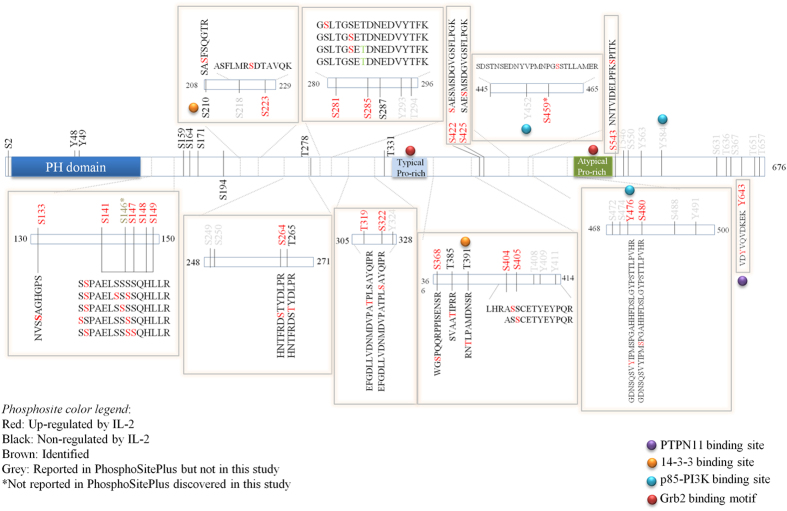
Gab2 phosphomap in IL-2-treated Kit225 T-lymphocytes. Schematic representation of Gab2 structure indicating motifs, phosphosites and protein binding sites. IL-2-dependent and –independent phosphosites reported in this study are highlighted in red and black, respectively. *indicates that the phosphosite is not reported in PhosphoSitePlus database.

Gab proteins can be recruited to the activated receptors through distinct mechanisms but according to the accumulated knowledge, the only mode for receptor interaction for Gab2 is indirectly via Grb2^16^. Grb2 is a small adaptor protein composed by a Src homology 2 (SH2) domain that allows association with phosphorylated tyrosine resides, flanked by two SH3 domains that recognize proline-rich motifs^47^. Depending on the cellular system Grb2/Gab2 complex has been found to be either constitutive or inducible. Whereas IL-3 stimulation of the murine pro-B-cell line Ba/F3 results in a significantly enhanced interaction between Grb2 and Gab2^48^, both proteins are identically associated in murine myeloid FDC-P1 cells treated or not with the colony-stimulating factor (M-CSF)^49^. We provide substantial evidence indicating that the adaptor protein Grb2 is constitutively associated with Gab2 in CD4^+^ Kit225 T-lymphocytes. Consequently, as the binding does not depend on tyrosine phosphorylation, it could be postulated that Grb2 SH3 domain and Gab2 proline-rich domains are involved in the formation of Grb2/Gab2 complex in Kit225 T-cells (Fig. 7). Noticeably, our previous study demonstrated that Grb2 can be recruited to cytokine-induced pY375 on IL-2Rγ (manuscript in preparation). Thus, it seem plausible that upon IL-2 stimulation Grb2 and its constitutive interactor Gab2 are recruited to the activated receptor complex through the SH2 domains of Grb2.

It has been demonstrated that Grb2/Gab2 interaction can be disrupted as a consequence of Gab2 S210 and T391 phosphorylation and subsequent 14-3-3 protein recruitment^27^. We show that Gab2 pS210 and pT391 are not robustly induced in response to cytokine stimulation. In accordance, our Gab2 interactome study demonstrates that 14-3-3 beta/alpha, which displayed a SILAC ratio of 1.4 (Supplementary Table S2), is not recruited to Gab2 in IL-2-treated T-lymphocytes. Moreover, we observe that Grb2/Gab2 interaction remains unaffected upon cytokine stimulation (Fig. 7). Overall, the integration of the quantitative proteomics and phosphoproteomics analyses indicate that this negative feedback mechanism is still not activated in Kit225 T-lymphocytes treated with IL-2 for 5 minutes.

Once assembled within the activated receptor complex, Gab2 is tyrosine phosphorylated in order to recruit downstream signaling molecules. Accordingly, our combinatorial Q-MS screen depicted several IL-2-triggered tyrosine phosphorylations in addition to numerous IL-2-inducible Gab2-interacting proteins. We demonstrate here that Gab2 Y476 and Y643 become phosphorylated *in vivo* upon 5 minutes treatment with IL-2 and provide substantial evidence indicating that the endogenous p85 regulatory subunit of PI3-kinase and tyrosine phosphatase PTPN11 associate with the scaffold protein in response to cytokine stimulation of T-lymphocytes. This is well in line with previous studies indicating that among others Gab2 pY476 and pY643 are involved in recruiting the p85 subunit of the PI3-kinase and the tyrosine phosphatase PTPN11, respectively^39^,^40^. Additionally, it has been reported that Gab2 pS210 inhibits the recruitment of the tyrosine phosphatase PTPN11 in response to growth factors^50^. As mentioned above, our study unveiled that Gab2 pS210 is not subjected to IL-2-mediated regulation after 5 minutes stimulation with the cytokine. These data suggest that the mechanisms disrupting the interaction between Gab2 and PTPN11 are not activated at early stages of IL-2 signal transduction.

Our investigation also resulted in the detection of three catalytic subunits of the Class IA PI3K family (PIK3CA, PIK3CB and PIK3CD) as cytokine-induced Gab2-binding proteins. In an attempt to determine whether they are direct or indirect interactors of the scaffold protein we calculated their relative abundance within the complex. In comparison to the well-known direct Gab2 interaction partners PTPN11 and p85 PI3-kinase that were found to be the most prominent interactors of the scaffold protein upon IL-2 stimulation, the relative abundance of the three catalytic PI3K subunits was very low. Hence, we postulate that whereas PTPN11 and p85 associate directly with tyrosine phosphorylated Gab2, the catalytic PI3K subunits detected in this study are recruited to the scaffold protein indirectly via the p85 regulatory subunit.

The formation of Gab2/PTPN11 and Gab2/PI3K complexes leads to the activation of RAS/MAPK and PI3K/AKT signaling branches, respectively. Both pathways are essential to assure IL-2-induced proliferation of Kit225 T-lymphocytes as we demonstrated in our previous study^20^. MAPK and AKT serine/threonine kinases are the two core representatives of RAS/MAPK and PI3K/AKT pathways and both are known to phosphorylate a plethora of substrates. In addition to targeting downstream signaling molecules, both kinases also phosphorylate upstream effectors creating negative feedback mechanisms that are fundamental for the adequate orchestration of signal transduction. Arnaud and colleagues demonstrated that pre-treatment of T-cells with MEK and PI3K inhibitor decreased the characteristic shift of Gab2 in response to IL-2 suggesting that both signaling branches are involved in phosphorylating the scaffold protein Gab2^17^. In fact, the study presented here uncovers the enormous impact IL-2 exerts on the serine/threonine phosphorylation status of Gab2. We found numerous site-specific Gab2 phosphorylations, including previously non-reported ones, that were induced in response to IL-2 stimulation in T-lymphocytes.

Recently, Eulenfeld and co-workers have shown that MAPK-dependent phosphorylation on S551 in Gab1 is crucial for the recruitment of the scaffold protein to the plasma membrane and initiate a positive-feedback loop that results in enhanced MAPK activation^51^. This specific Gab1 residue is conserved in Gab2 and corresponds to S543. Strikingly, our SILAC-based quantitative phosphoproteomics screen shows that Gab2 pS543 is induced *in vivo* in Kit225 T-lymphocytes. Thus, it is likely that Gab2 pS543 is also involved in the membrane recruitment of Gab2 which allows the scaffold protein being in close proximity with signaling effectors that assure the propagation of signal transduction.

More importantly, this study reveals large number of Gab2 serine/threonine phosphorylations, including three novel phosphorylated serines, that are modulated upon IL-2 stimulation of CD4^+^ T-lymphocytes (Fig. 7). In addition, we also discovered and further validated that DLAT, ARID3A and ARID3B associate with Gab2 in response to cytokine stimulation in Kit225 T-cells. The overall regulation of Gab2-mediated signal transduction is determined by the crosstalk of phosphosites that ultimately orchestrate the protein recruitment/release balance. Consequently, this study provides a wealth of candidates for future hypothesis-driven mechanism-focused studies that may gain insights into the regulation and role of Gab2 in the signal propagation initiated by IL-2.

## Methods

### Cell culture and stimulation

The IL-2-dependent human T-cell line Kit225^52^ was maintained in RPMI 1640 medium supplemented with 10% FBS, 1% Glutamax, 1% penicillin/streptomycin, 1% sodium pyruvate and 16 U/ml of recombinant human IL-2 (kindly provided by AIDS Research and Reference Reagent Program, Division of AIDS, NIH, USA) at a density of 1 × 10^6^ cells/ml at 37 °C and 5% CO_2_. For SILAC experiments the RPMI media was custom-made and deficient for L-Arg, and L-Lys (Gibco-Invitrogen). It was supplemented with glutamax, p/s and sodium pyruvate as mentioned before together with 10% dialyzed serum (Gibco-Invitrogen) and different versions of L-Lys (Lys0 or Lys4) and L-Arg (Arg0 or Arg6) from Cambridge Isotope Laboratories.

Prior stimulation Kit225 T-cells were IL-2-deprived for 48 h to arrest them at the G_1_ phase of the cell cycle and resemble resting T-cells. IL-2-dependent signaling networks were induced by adding 200 U of IL-2 to 50.10^6^ cells/ml and incubating for 5 min at 37 °C. In SILAC experiments, Kit225 T-cells grown in light media (Arg0/Lys0) were kept unstimulated and served as control whereas cells grown in heavy media (Arg6/Lys4) were stimulated with the cytokine as described above. Treatment was quenched by keeping cells on ice for 5 min and rapidly washing them repeatedly with chilled PBS prior proceeding with cell lysis.

Human cervix epithelial adenocarcinoma HeLa cells were grown in Dulbecco’s modified Eagle’s medium (DMEM, Lonza) supplemented with 10% Fetal Bovine Serum, 1% Penicillin-Streptomycin and 1% L-Glutamine.

### Protein extraction and immunoprecipitation

Kit225 T-cells were lysed with ice-cold co-immunopreicpitation buffer (25 mM TrisHCl pH 7.5, 100 mM NaCl, 1% NP-40, 1 mM sodium pervanadate, 5 mM beta-glycerophosphate, 5 mM NaF, complete protease inhibitor cocktail (complete tablets, Roche)) and protein concentrations were estimated using BCA protein assay kit (Pierce).

In SILAC experiments protein lysates corresponding to untreated and IL-2-stimulated cells were combined in 1:1 ratio according to their protein concentration whereas in non-SILAC experiments differentially treated cell conditions were processed separately. In both cases, cell lysates were pre-cleared for 1 h at 4 °C while magnetic beads coupled with protein G (ThermoFischer Scientific) were loaded with the antibody recognizing Gab2 by incubation at 4 °C. For immunoprecipitating Gab2 together with its corresponding binding partners, pre-cleared lysates were incubated with the magnetic beads coupled with the Gab2 antibody for 2 h at 4 °C in rotation. Then, immunoprecipitated proteins were washed 3 times with lysis buffer and eluted by 5 min boiling with loading buffer (50 mM Tris pH 6.8, 5% glycerol, 1.67% beta-mercaptoethanol, 1.67% SDS).

### In-gel digestion and peptide extraction

Eluted immunecomplexes were run in two parallel lanes of a precast NuPAGE 4–12% Bis-Tris Gel (Invitrogen) and visualized with Colloidal Blue (Invitrogen). Both gel lanes were separately cut into slices and subjected to in-gel trypsinization (Promega) as previously described^53^. Briefly, gel slices were subjected to reduction with 10 mM DTT, alkylation by 55 mM chloroacetamide and protein digestion by incubating with trypsin overnight at 37 °C.

Resulting tryptic peptides were extracted from the gel by serial incubations with 100% ACN and 30% ACN/3% TFA. Finally, the solutions obtained in all the incubations were pooled together and dried down in a vacuum centrifuge. Whereas peptides derived from one of the lanes were directly concentrated and desalted using C_18_ stage tips (made in house using Empore disc –C18 Agilent Life Science) to further analyze by LC-MS/MS, peptides derived from the other lane were subjected to TiO_2_ enrichment prior to MS analysis.

### Enrichment of phosphopeptides using TiO_2_ beads

Enrichment of phosphopeptides was performed as previously described^53^,^54^. 5 mm diameter titansphere beads (GL Sciences) were mixed with the equilibration buffer (50 mg/ml DHB in 80%ACN/1%TFA) 1:1 ratio (w/w) and incubated for 30 min at room temperature whereas vacuum-dried samples were adjusted to 60% ACN/1% TFA. 2 μl of equilibrated TiO_2_ slurry was added to the sample and incubated for 30 min at room temperature in with rotation for allowing phosphopeptides bind the beads. After brief centrifugation, supernatant was removed and TiO_2_ beads were transferred onto a home-made C_8_ Stage-Tip (Empore disc –C8 Agilent Life Science) where they were washed twice with 60% ACN/1% TFA. Phosphopeptides were eluted from the beads by two subsequent incubations using 5% NH_4_OH and 25% NH_4_OH/0.3% TFA. Finally, eluted phosphopeptides were dried down in a vacuum centrifuge and desalted using C_18_ stage tips prior LC-MS/MS analysis.

### LC-MS/MS and data analysis

LC-MS/MS analysis of was carried out using a reverse phase liquid chromatography system (EASY-nLC 1000 ultra-high pressure, Thermo Fisher Scientific) interfaced with a Q Exactive mass spectrometer (Thermo Fischer Scientific) via a nanoelectrospray source (Thermo Fisher Scientific). Acidified peptides were loaded on an analytical in-house packed column (20 cm × 75 μm, ReproSil-Pur C_18_-AQ 3 μm resin (Dr. Maisch GmbH)) in solvent A (0.5% acetic acid) and eluted by a nonlinear 120 min solvent B gradient (0.5% acetic acid, 80% ACN) at a flow rate of 250 nL/min. Q Exactive was operated in a top 10 data dependent mode. Survey scans were acquired at a resolution of 70,000 (m/z 400) and fragmentation spectra at 35,000 (m/z 400). Precursors were fragmented by higher energy C-trap dissociation (HCD) with normalized collision energy of 25 eV. The maximum injection time was 120 ms for survey and 124 ms for MS/MS scan. Repeat sequencing of peptide was minimized by excluding the selected peptide candidates for 45 s.

All raw data files acquired were searched against the UniProt human database version 2014.01 (with 88,479 sequence entries) with MaxQuant proteomics computational platform version 1.3.0.5 and using Andromeda search engine^55^. In SILAC experiments light and heavy labels were set as Arg0/Lys0 and Arg6/Lys4. Precursor and fragment mass tolerances were set to 7 and 20 ppm, respectively. Enzyme specificity was set to trypsin, allowing for cleavage N-terminal to proline and between aspartic acid and proline with a maximum of 2 missed cleavages. Carbamidomethylation of cysteine (C) was set as fixed modification whereas oxidation of methionine (M), protein N-terminal acetylation, deamidation of asparagine and glutamine (NQ) and phosphorylation of serine/threonine/tyrosine (STY) were selected as variable modifications for database searching. For peptide and protein identification a minimal peptide length of 7 amino acids was required and the false discovery rate was set at 0.01. Additionally, for protein identification we demanded at least two peptides, of which at least one was unique to the protein group. Both razor and unique peptides, except STY phosphorylated peptides were considered for protein quantification. For the analysis of phosphopeptides, 1% FDR, a minimum localization probability of 0.75 and a score difference of at least 5 was used^56^. Proteins with ratio IL-2/Ctr > 1.5; Significance B < 0.0001 were regarded as significantly changing in the immunoprecipitated complexes following IL-2 treatment. In respect to phosphorylation changes, ratios IL-2/Ctr > 2 were considered as significant.

The mass spectrometry proteomics data have been deposited to the ProteomeXchange Consortium^57^ via PRIDE partner repository with the dataset identifier PXD003286.

### Bioinformatic analysis

Perseus statistical software was employed for the calculation of the statistical significance B, a p-value that depends on protein intensities and ratios. It was also used to perform the heat map and hierarchical clustering analysis in which protein groups were clustered according to their ratios and following next settings: row and column distance calculated using the Euclidean algorithm; row and column linkage – average^58^. Phosphosites not reported in the PhosphoSitePlus database (http://www.phosphosite.org/homeAction.action) were considered as novel. MS/MS spectra of the phosphorylated peptides bearing novel phosphosites of Gab2 were validated and annotated using MaxQuant viewer expert system^59^. Protein sequence alignment was performed by SIM (http://web.expasy.org/sim/) available in ExPASy Bioinformatics Resource Portal.

### Western blot

Eluted immune complexes were resolved in precast gradient NuPAGE 4–12% Bis-Tris gels (Invitrogen) and transferred onto nitrocellulose membranes (Bio-Rad). Membranes were blocked with 2% BSA and incubated overnight at 4 °C with primary antibodies, including; Gab2 (sc-9313), SHP2 (sc-7844) and 14-3-3 (sc-25276) from SantaCruz Biotechnology, ARID3A (A300-228 A) and ARID3B (A302-564 A) from Methyl Lab, GRB2 (610111, Transduction Lab), PI3 kinase p85 (#4257, Cell Signaling) and DLAT (ab126224, Abcam). Then, the membranes were repeatedly washed with TBST buffer (150 mM NaCl, 10 mM Tris, pH 8.0 and 0.1% Tween 20) and incubated with HRP-conjugated anti-rabbit (NA934, GE Healthcare), anti-mouse (NA931, GE Healthcare) or anti-goat (ref) secondary antibodies for 1 h at room temperature. Finally, the membranes were washed and processed with ECL solution (PRN2106, GE Healthcare) for detection scanning on a Kodak Image Station 1000. The intensity of the bands was quantified using ImageJ software (National Institutes of health, Bethesda, MD, USA).

### RNA isolation and real-time qPCR for gene expression analyses

Total RNA was extracted from cells using TRIzol reagent (Invitrogen) following manufacturer’s instructions. 1 μg of RNA was then reverse transcribed into cDNA by incubating with a reaction mixture containing 0.25 μg of random hexamers and 1 mM dNTP mix for 5 min at 65 °C followed by 1 h incubation at 37 °C with 1^st^ Stand buffer (Invitrogen), 1 mM DTT and 12 U of M-MLV reverse transcriptase (Promega). cDNA was amplified in the Strategene Mx2000P System using SYBR Green JumpStart Taq Ready Mix (Sigma-Aldrich) and quantified using Stratagene MxPro. Amplification of β-actin was used for normalization. Sequences of PCR primers are listed in Supplementary Table S1.

## Additional Information

**How to cite this article**: Osinalde, N. *et al.* Changes in Gab2 phosphorylation and interaction partners in response to interleukin (IL)-2 stimulation in T-lymphocytes. *Sci. Rep.* **6**, 23530; doi: 10.1038/srep23530 (2016).

## Supplementary Material

Supplementary Information

Supplementary Datset 1

## Figures and Tables

**Table 1:** Gab2 serine/threonine phosphorylations.

ModifiedGab2 site	Modified sequence	PEP	Score	Charge	m/z	Replica 1			Replica 2			Conserved in Gab3
						IL-2/Ctrratio	Peak Intensity		IL-2/Ctrratio	Peak Intensity		
							Ctr	IL-2		Ctr	IL-2	
S133	NVSpSAGHGPR	3.9E-03	123.92	2	531.23	27.0	3.1E+06	8.6E+07	–	–	–	S132*
S141	SpSPAELSSSSQHLLR	3.9E-98	253.73	2	839.89	9.0	1.1E+09	2.5E+09	3.5	1.5E+09	2.5E+09	T134*
S146*	SpSPAELpSSpSSQHLLR	1.8E-02	61.181	2	919.86	–	–	–	–	–	–	no
S147*	SpSPAELSpSSSQHLLR	8.0E-48	183.56	2	879.88	–	–	2.7E+07	31.1	4.2E+05	1.7E+07	no
S148	pSSPAELSSpSSQHLLR	3.9E-98	259.18	2	879.88	7.0	3.0E+07	8.6E+08	2.8	4.3E+07	6.5E+08	S141*
S149	SpSPAELSSpSpSQHLLR	1.0E-72	259.18	2	919.86	–	–	1.0E+06	–	–	2.1E+06	S142*
*S210*	SApSFSQGTR	9.6E-05	183.99	2	510.71	2.0	4.3E+09	6.7E+09	1.9	1.6E+09	2.3E+09	no
S223	ASFLoxMRpSDTAVQK	7.9E-21	184.37	2	775.36	8.1	6.2E+08	1.2E+09	2.2	6.4E+08	7.3E+08	S209*
S264	HNTEFRDpSTYDLPR	5.9E-84	269.71	3	610.93	4.3	4.5E+08	1.7E+09	9.9	7.9E+07	3.1E+08	no
T265	HNTEFRDSpTYDLPR	2.9E-02	89.353	2	915.89	–	–	–	–	–	–	no
S281	GpSLTGSETDNEDVYTFK	5.8E-77	256.11	2	971.90	–	–	3.7E+07	3.8	2.9E+06	3.2E+07	S242*
S285	GSLTGpSETDNEDVYTFK	2.4E-52	298.22	2	971.90	3.4	5.9E+07	4.4E+08	3.9	4.5E+07	2.8E+08	no
*T287*	GSLTGSEpTDNEDVYTFK	6.3E-83	375.45	2	971.90	0.5	3.1E+08	2.1E+08	0.6	2.0E+08	1.3E+08	no
T319	EFGDLLVDNoxMDVPApTPLSAYQIPR	6.7E-29	184	2	1379.15	7.9	9.9E+06	3.2E+08	18.8	1.9E+06	9.2E+07	S273*
S322	EFGDLLVDNoxMDVPATPLpSAYQIPR	1.5E-20	170.27	3	919.77	9.1	3.2E+06	8.5E+07	10.3	1.7E+06	6.3E+07	no
S368	WGpSPQQRPPISENSR	2.3E-02	68.567	3	606.95	2.3	4.3E+08	2.7E+08	–	–	–	no
*T385*	SVAApTIPRR	1.2E-04	128.01	2	525.78	0.5	3.0E+08	1.0E+08	1.1	1.2E+08	4.2E+07	no
*T391*	RNpTLPAMDNSR	1.2E-13	265.64	3	452.20	1.5	1.5E+09	2.8E+09	1.3	3.8E+08	4.0E+08	S344
S404	LHRApSSCETYEYPQR	3.8E-06	141.6	3	659.62	13.8	7.9E+05	3.1E+07	5.1	2.2E+05	1.9E+06	no
S405	ASpSCETYEYPQR	6.0E-07	137.81	2	785.80	8.0	1.1E+07	9.9E+07	2.0	4.7E+07	4.7E+07	no
S422	pSAESoxMSDGVGSFLPGK	1.3E-44	210.75	2	832.85	8.3	4.4E+06	3.4E+07	5.0	3.4E+07	6.3E+07	S362
S425	SAEpSMSDGVGSFLPGK	9.7E-45	221.79	2	824.85	5.9	4.4E+07	1.8E+08	7.1	1.6E+07	1.1E+08	no
S459*	SDSTNSEDNYVPoxMNPGpSSTLLAoxMER	2.7E-12	143.07	2	1414.08	14.5	3.1E+06	1.6E+08	7.5	2.9E+06	1.1E+08	no
S480	AGDNSQSVYIPoxMpSPGAHHFDSLGYPSTTLPVHR	1.3E-57	197	4	909.42	2.2	5.7E+08	1.7E+09	2.2	5.6E+08	1.1E+09	no
S543	NNTVIDELPFKpSPITK	1.0E-210	350.82	2	948.48	8.7	5.0E+08	5.1E+09	4.9	4.8E+08	2.0E+09	no

In bold are phosphorylations induced by IL-2 whereas IL-2-independent phosphorylations are in italics. *indicates that the phosphorylated site is not reported in PhosphoSitePlus database.

## References

[b1] Boyman O., Cho J. H. & Sprent J. The role of interleukin-2 in memory CD8 cell differentiation. Adv Exp Med Biol 684, 28–41 (2010). 20795538 10.1007/978-1-4419-6451-9_3

[b2] Kamimura D. & Bevan M. J. Naive CD8^+^ T cells differentiate into protective memory-like cells after IL-2 anti IL-2 complex treatment *in vivo*. J Exp Med 204, 1803–1812, doi: 10.1084/jem.20070543 (2007). 17664293 PMC2118678

[b3] Liao W., Lin J. X., Wang L., Li P. & Leonard W. J. Modulation of cytokine receptors by IL-2 broadly regulates differentiation into helper T cell lineages. Nat Immunol 12, 551–559, doi: 10.1038/ni.2030 (2011). 21516110 PMC3304099

[b4] Williams M. A., Tyznik A. J. & Bevan M. J. Interleukin-2 signals during priming are required for secondary expansion of CD8^+^ memory T cells. Nature 441, 890–893, doi: 10.1038/nature04790 (2006). 16778891 PMC2776073

[b5] Restifo N. P., Dudley M. E. & Rosenberg S. A. Adoptive immunotherapy for cancer: harnessing the T cell response. Nat Rev Immunol 12, 269–281, doi: 10.1038/nri3191 (2012). 22437939 PMC6292222

[b6] Rosenberg S. A. IL-2: the first effective immunotherapy for human cancer. J Immunol 192, 5451–5458, doi: 10.4049/jimmunol.1490019 (2014). 24907378 PMC6293462

[b7] Dybkaer K. *et al.* Genome wide transcriptional analysis of resting and IL2 activated human natural killer cells: gene expression signatures indicative of novel molecular signaling pathways. Bmc Genomics 8, doi: Artn23010.1186/1471-2164-8-230 (2007). 10.1186/1471-2164-8-230 PMC1959522 17623099

[b8] Friedman M. C., Migone T. S., Russell S. M. & Leonard W. J. Different interleukin 2 receptor beta-chain tyrosines couple to at least two signaling pathways and synergistically mediate interleukin 2-induced proliferation. Proc Natl Acad Sci USA 93, 2077–2082, doi: 10.1073/pnas.93.5.2077 (1996). 8700888 PMC39912

[b9] Nelson B. H., Lord J. D. & Greenberg P. D. A membrane-proximal region of the interleukin-2 receptor gamma(c) chain sufficient for Jak kinase activation and induction of proliferation in T cells. Mol Cell Biol 16, 309–317 (1996). 8524310 10.1128/mcb.16.1.309 PMC231005

[b10] Usacheva A. *et al.* Contribution of the Box 1 and Box 2 motifs of cytokine receptors to Jak1 association and activation. J Biol Chem 277, 48220–48226, doi: 10.1074/jbc.M205757200 (2002). 12374810

[b11] Okutani Y. *et al.* Src directly tyrosine-phosphorylates STAT5 on its activation site and is involved in erythropoietin-induced signaling pathway. Oncogene 20, 6643–6650, doi: 10.1038/sj.onc.1204807 (2001). 11641791

[b12] Zhou Y. J. *et al.* Distinct tyrosine phosphorylation sites in JAK3 kinase domain positively and negatively regulate its enzymatic activity. Proc Natl Acad Sci USA 94, 13850–13855 (1997). 9391116 10.1073/pnas.94.25.13850 PMC28396

[b13] Shaw A. S. & Filbert, E. L. Scaffold proteins and immune-cell signalling. Nat Rev Immunol 9, 47–56, doi: 10.1038/nri2473 (2009). 19104498

[b14] Halbach S. *et al.* Alterations of Gab2 signalling complexes in imatinib and dasatinib treated chronic myeloid leukaemia cells. Cell Commun Signal 11, 30, doi: 10.1186/1478-811X-11-30 (2013). 23607741 PMC3640961

[b15] Nishida K. *et al.* Gab-family adapter proteins act downstream of cytokine and growth factor receptors and T- and B-cell antigen receptors. Blood 93, 1809–1816 (1999). 10068651

[b16] Wohrle F. U., Daly R. J. & Brummer T. Function, regulation and pathological roles of the Gab/DOS docking proteins. Cell Commun Signal 7, 22, doi: 10.1186/1478-811X-7-22 (2009). 19737390 PMC2747914

[b17] Arnaud M., Crouin C., Deon C., Loyaux D. & Bertoglio J. Phosphorylation of Grb2-associated binder 2 on serine 623 by ERK MAPK regulates its association with the phosphatase SHP-2 and decreases STAT5 activation. J Immunol 173, 3962–3971 (2004). 15356145 10.4049/jimmunol.173.6.3962

[b18] Arneja A., Johnson H., Gabrovsek L., Lauffenburger D. A. & White F. M. Qualitatively different T cell phenotypic responses to IL-2 versus IL-15 are unified by identical dependences on receptor signal strength and duration. J Immunol 192, 123–135, doi: 10.4049/jimmunol.1302291 (2014). 24298013 PMC3950894

[b19] Gadina M., Sudarshan C. & O’Shea J. J. IL-2, but not IL-4 and other cytokines, induces phosphorylation of a 98-kDa protein associated with SHP-2, phosphatidylinositol 3′-kinase, and Grb2. J Immunol 162, 2081–2086 (1999). 9973481

[b20] Osinalde N. *et al.* Interleukin-2 signaling pathway analysis by quantitative phosphoproteomics. J Proteomics 75, 177–191, doi: 10.1016/j.jprot.2011.06.007 (2011). 21722762

[b21] Osinalde N. *et al.* Simultaneous dissection and comparison of IL-2 and IL-15 signaling pathways by global quantitative phosphoproteomics. Proteomics 15, 520–531, doi: 10.1002/pmic.201400194 (2015). 25142963

[b22] Liu Y. & Rohrschneider L. R. The gift of Gab. FEBS Lett 515, 1–7 (2002). 11943184 10.1016/s0014-5793(02)02425-0

[b23] Adams S. J., Aydin I. T. & Celebi J. T. GAB2–a scaffolding protein in cancer. Mol Cancer Res 10, 1265–1270, doi: 10.1158/1541-7786.MCR-12-0352 (2012). 22871571 PMC3810274

[b24] Brummer T. *et al.* Increased proliferation and altered growth factor dependence of human mammary epithelial cells overexpressing the Gab2 docking protein. J Biol Chem 281, 626–637, doi: 10.1074/jbc.M509567200 (2006). 16253990

[b25] Daly R. J. *et al.* The docking protein Gab2 is overexpressed and estrogen regulated in human breast cancer. Oncogene 21, 5175–5181, doi: 10.1038/sj.onc.1205522 (2002). 12140767

[b26] Lynch D. K. & Daly R. J. PKB-mediated negative feedback tightly regulates mitogenic signalling via Gab2. EMBO J 21, 72–82 (2002). 11782427 10.1093/emboj/21.1.72 PMC125816

[b27] Brummer T. *et al.* Phosphorylation-dependent binding of 14-3-3 terminates signalling by the Gab2 docking protein. EMBO J 27, 2305–2316 (2008). 19172738 10.1038/emboj.2008.159 PMC2529372

[b28] Ortiz-Padilla C. *et al.* Functional characterization of cancer-associated Gab1 mutations. Oncogene 32, 2696–2702, doi: 10.1038/onc.2012.271 (2013). 22751113

[b29] Blagoev B. *et al.* A proteomics strategy to elucidate functional protein-protein interactions applied to EGF signaling. Nat Biotechnol 21, 315–318, doi: 10.1038/nbt790nbt790 (2003). 12577067

[b30] Chylek L. A. *et al.* Phosphorylation site dynamics of early T-cell receptor signaling. PLoS One 9, e104240, doi: 10.1371/journal.pone.0104240PONE-D-14-22902 (2014). 25147952 PMC4141737

[b31] Dengjel J., Kratchmarova I. & Blagoev B. Receptor tyrosine kinase signaling: a view from quantitative proteomics. Mol Biosyst 5, 1112–1121, doi: 10.1039/b909534a (2009). 19756300

[b32] Dengjel J., Kratchmarova I. & Blagoev B. Mapping protein-protein interactions by quantitative proteomics. Methods Mol Biol 658, 267–278, doi: 10.1007/978-1-60761-780-8_16 (2010). 20839110

[b33] Hein M. Y. *et al.* A human interactome in three quantitative dimensions organized by stoichiometries and abundances. Cell 163, 712–723, doi: S0092-8674(15)01270-210.1016/j.cell.2015.09.053 (2015). 26496610 10.1016/j.cell.2015.09.053

[b34] Seiffert M. *et al.* Gab3-deficient mice exhibit normal development and hematopoiesis and are immunocompetent. Mol Cell Biol 23, 2415–2424 (2003). 12640125 10.1128/MCB.23.7.2415-2424.2003 PMC150735

[b35] Wada T. *et al.* The molecular scaffold Gab2 is a crucial component of RANK signaling and osteoclastogenesis. Nat Med 11, 394–399, doi: 10.1038/nm1203 (2005). 15750601

[b36] Zhang Y. *et al.* Abnormal hematopoiesis in Gab2 mutant mice. Blood 110, 116–124, doi: 10.1182/blood-2006-11-060707 (2007). 17374739 PMC1896106

[b37] Wilson-Grady J. T., Haas W. & Gygi S. P. Quantitative comparison of the fasted and re-fed mouse liver phosphoproteomes using lower pH reductive dimethylation. Methods 61, 277–286, doi: 10.1016/j.ymeth.2013.03.031 (2013). 23567750

[b38] Lewitzky M. *et al.* The C-terminal SH3 domain of the adapter protein Grb2 binds with high affinity to sequences in Gab1 and SLP-76 which lack the SH3-typical P-x-x-P core motif. Oncogene 20, 1052–1062, doi: 10.1038/sj.onc.1204202 (2001). 11314042

[b39] Arnaud M. *et al.* Interaction of the tyrosine phosphatase SHP-2 with Gab2 regulates Rho-dependent activation of the c-fos serum response element by interleukin-2. Biochem J 382, 545–556, doi: 10.1042/BJ20040103 (2004). 15170389 PMC1133811

[b40] Crouin C., Arnaud M., Gesbert F., Camonis J. & Bertoglio J. A yeast two-hybrid study of human p97/Gab2 interactions with its SH2 domain-containing binding partners. FEBS Lett 495, 148–153 (2001). 11334882 10.1016/s0014-5793(01)02373-0

[b41] Gesbert F., Guenzi C. & Bertoglio J. A new tyrosine-phosphorylated 97-kDa adaptor protein mediates interleukin-2-induced association of SHP-2 with p85-phosphatidylinositol 3-kinase in human T lymphocytes. J Biol Chem 273, 18273–18281 (1998). 9660791 10.1074/jbc.273.29.18273

[b42] Silva J. C., Gorenstein M. V., Li G. Z., Vissers J. P. & Geromanos S. J. Absolute quantification of proteins by LCMSE: a virtue of parallel MS acquisition. Mol Cell Proteomics 5, 144–156, doi: 10.1074/mcp.M500230-MCP200 (2006). 16219938

[b43] Johnston J. A. *et al.* Tyrosine phosphorylation and activation of STAT5, STAT3, and Janus kinases by interleukins 2 and 15. Proc Natl Acad Sci USA 92, 8705–8709 (1995). 7568001 10.1073/pnas.92.19.8705 PMC41035

[b44] Nakaoka Y. & Komuro I. Gab docking proteins in cardiovascular disease, cancer, and inflammation. Int J Inflam 2013, 141068, doi: 10.1155/2013/141068 (2013). 23431498 PMC3566608

[b45] Vaughan T. Y., Verma S. & Bunting K. D. Grb2-associated binding (Gab) proteins in hematopoietic and immune cell biology. Am J Blood Res 1, 130–134 (2011). 22163099 PMC3232456

[b46] Holgado-Madruga M., Emlet D. R., Moscatello D. K., Godwin A. K. & Wong A. J. A Grb2-associated docking protein in EGF- and insulin-receptor signalling. Nature 379, 560–564, doi: 10.1038/379560a0 (1996). 8596638

[b47] Reebye V. *et al.* A perspective on non-catalytic Src homology (SH) adaptor signalling proteins. Cell Signal 24, 388–392, doi: 10.1016/j.cellsig.2011.10.003 (2012). 22024281

[b48] Nyga R. *et al.* Activated STAT5 proteins induce activation of the PI 3-kinase/Akt and Ras/MAPK pathways via the Gab2 scaffolding adapter. Biochem J 390, 359–366, doi: 10.1042/BJ20041523 (2005). 15833084 PMC1188271

[b49] Liu Y., Jenkins B., Shin J. L. & Rohrschneider L. R. Scaffolding protein Gab2 mediates differentiation signaling downstream of Fms receptor tyrosine kinase. Mol Cell Biol 21, 3047–3056, doi: 10.1128/MCB.21.9.3047-3056.2001 (2001). 11287610 PMC86933

[b50] Zhang X. *et al.* Gab2 phosphorylation by RSK inhibits Shp2 recruitment and cell motility. Mol Cell Biol 33, 1657–1670, doi: 10.1128/MCB.01353-12 (2013). 23401857 PMC3624252

[b51] Eulenfeld R. & Schaper F. A new mechanism for the regulation of Gab1 recruitment to the plasma membrane. J Cell Sci 122, 55–64, doi: 10.1242/jcs.037226 (2009). 19050043

[b52] Hori T. *et al.* Establishment of an interleukin 2-dependent human T cell line from a patient with T cell chronic lymphocytic leukemia who is not infected with human T cell leukemia/lymphoma virus. Blood 70, 1069–1072 (1987). 3115332

[b53] Osinalde N., Sanchez-Quiles V., Akimov V., Blagoev B. & Kratchmarova I. SILAC-based quantification of changes in protein tyrosine phosphorylation induced by Interleukin-2 (IL-2) and IL-15 in T-lymphocytes. Data Brief 5, 53–58, doi: 10.1016/j.dib.2015.08.007 (2015). 26425665 PMC4564383

[b54] Larsen M. R., Thingholm T. E., Jensen O. N., Roepstorff P. & Jorgensen T. J. Highly selective enrichment of phosphorylated peptides from peptide mixtures using titanium dioxide microcolumns. Mol Cell Proteomics 4, 873–886, doi: 10.1074/mcp.T500007-MCP200 (2005). 15858219

[b55] Cox J. *et al.* Andromeda: a peptide search engine integrated into the MaxQuant environment. J Proteome Res 10, 1794–1805, doi: 10.1021/pr101065j (2011). 21254760

[b56] Olsen J. V. *et al.* Global, *in vivo*, and site-specific phosphorylation dynamics in signaling networks. Cell 127, 635–648, doi: 10.1016/j.cell.2006.09.026 (2006). 17081983

[b57] Vizcaino J. A. *et al.* ProteomeXchange provides globally coordinated proteomics data submission and dissemination. Nat Biotechnol 32, 223–226, doi: 10.1038/nbt.2839 (2014). 24727771 PMC3986813

[b58] Cox J. & Mann M. MaxQuant enables high peptide identification rates, individualized p.p.b.-range mass accuracies and proteome-wide protein quantification. Nat Biotechnol 26, 1367–1372, doi: 10.1038/nbt.1511 (2008). 19029910

[b59] Neuhauser N., Michalski A., Cox J. & Mann M. Expert system for computer-assisted annotation of MS/MS spectra. Mol Cell Proteomics 11, 1500–1509, doi: 10.1074/mcp.M112.020271 (2012). 22888147 PMC3494176

