# Supplementary material for: Changes in Gab2 phosphorylation and interaction partners in response to interleukin (IL)-2 stimulation in T-lymphocytes
Source: Sci Rep. 2016 Mar 30;6:23530. doi: 10.1038/srep23530 (PMC4812247; doi:10.1038/srep23530)
Supplement: Supplementary Information [file srep23530-s1.pdf]

Supplementary Information

Changes in Gab2 phosphorylation and interaction partners in response to interleukin (IL)-2 stimulation in T-lymphocytes

Nerea Osinalde, Virginia Sánchez-Quiles, Blagoy Blagoev & Irina Kratchmarova*

Department of Biochemistry and Molecular Biology, University of Southern Denmark, 5230 Odense M, Denmark.

Osinalde_Supplementary Figure S1

```
33.4% identity in 668 residues overlap; Score: 475.0; Gap frequency: 13.8%

GAB2            5 GDVVCTGWLRKSPPEKKLRRYAWKKRWFILRSGRMSGDPDVLEYYKNDHSKKPLRIINLN
GAB3            4 GDAVCTGWLVKSPPERKLQRYAWRKRWFVLRRGRMSGNPDVLEYYRNKHSSKPIRVIDLS
                  ** ****** ***** ** **** **** ** ***** ******* * ** ** * * *

GAB2           65 FCEQ-VDAGLTFNKKELQDSFVFDIKTSERTFYLVAETEEDMNKWVQSICQICGFNQAEE
GAB3           64 ECAVWKHVGPSFVRKEFQNNFVFIVKTTSRTFYLVAKTEQEMQVWVHSISQVCNLGHLED
                   *      *  *  ** *  ***  **  ******* **  *  ** ** * *     *

GAB2          124 STDSLRNVSSAGHGPRSSPAELSSSSQHLLRERKSSAPSHSSQPTLFTFEPPVSNHMQPT
GAB3          124 GADSMESLSY-------TPSSLQPSSA------SSLLTAHAASSSLPRDDP--NTNAVAT
                    **    *         *  *  **        *    *     *    *        *

GAB2          184 LSTSAPQEYLYLHQ-CISRRAENARSASFSQGTRASFLMRSDTAVQKLAQGNGHCVNGIS
GAB3          169 EETRSESELLFLPDYLVLSNCETGRLHHTSLPTRCDSWSNSDRSLEQAS-----------
                    *    * * *         *  *    *  **      **

GAB2          243 GQVHGFYSLPKPSRHNTEFRDSTYDLPRSLASHGHTKGSLTGSETDNEDVYTFKTPSNTL
GAB3          218 --------------FDDVFVDCLQPLPSSHLVHPSCHGS--GAQ---EVPSSRPQAALIW
                                    * *    ** *   *    **  *     *

GAB2          303 CREFGDLLVDNMDVPATPLSAYQIPRTFTLDKNHNAMTVATPGDSAIA--PPPRPPKPSQ
GAB3          259 SREINGPPRDHLS--SSPLLESSLSSTIQVDKNQGSLPCGAKELDIMSNTPPPRPPKPSH
                   **      *       **      *   ***                 *********

GAB2          361 AETPRWGSPQQRPPISENSRSVAATIPRRNTLPAMDNSRLHRASSCETYEYPQRGGESAG
GAB3          317 LSERR---QEEWSTHSGSKKPECTLVPRRISLSGLDNMRTWKA-------------DVEG
                      *          *          ***  *   ** *   *                *

GAB2          421 RSAESMSDGVGSFLPGKMI-VGRSDSTNSEDNYVPMNPGSSTLLAMERAGDNSQSVYIPM
GAB3          361 QSLRHRDKRLSLNLPCRFSPMYPTASASIEDSYVPMSPQAG---ASGLGPHCSPDDYIPM
                   *           **          *   ** **** *      *       *   ****

GAB2          480 SPGAHHFDSLGYPSTTLPVHRGPSRGSEIQPPPVNRNLKPDRKAKPTPLDLRNNTVIDEL
GAB3          418 NSG-----SISSPLPELPAN--------LEPPPVNRDLKPQRKSRPPPLDLRNLSIIRE-
                    *     *   *   **            ****** *** **  * ******   * *

GAB2          540 PFKSPITKSWSRANHTFNSSSSQYCRPISTQSITSTDSGDSEENYVPMQNPVSASPVPSG
GAB3          464 --HASLTRTRTVPCSRTSFLSPERNGINSARFFANPVSREDEESYIEMEEHRTASSLSSG
                        *             *       *        *   ** *  *     **   **

GAB2          600 TNSPAPKKSTGSVDYLALDFQPSSPSPHRKPSTSSVTSDEKVDYVQVDKEKTQALQNTMQ
GAB3          522 ALTWTKK---FSLDYLALDFNSASPAPMQQKLL--LSEEQRVDYVQVDEQKTQALQSTKQ
                        *    * *******   ** *              *******  ****** * *

GAB2          660 EWTDVRQS
GAB3          577 EWTDERQS
```

Supplementary Figure S1. Sequence alignment between human Gab2 and Gab3.

Osinalde_Supplementary Figure S2

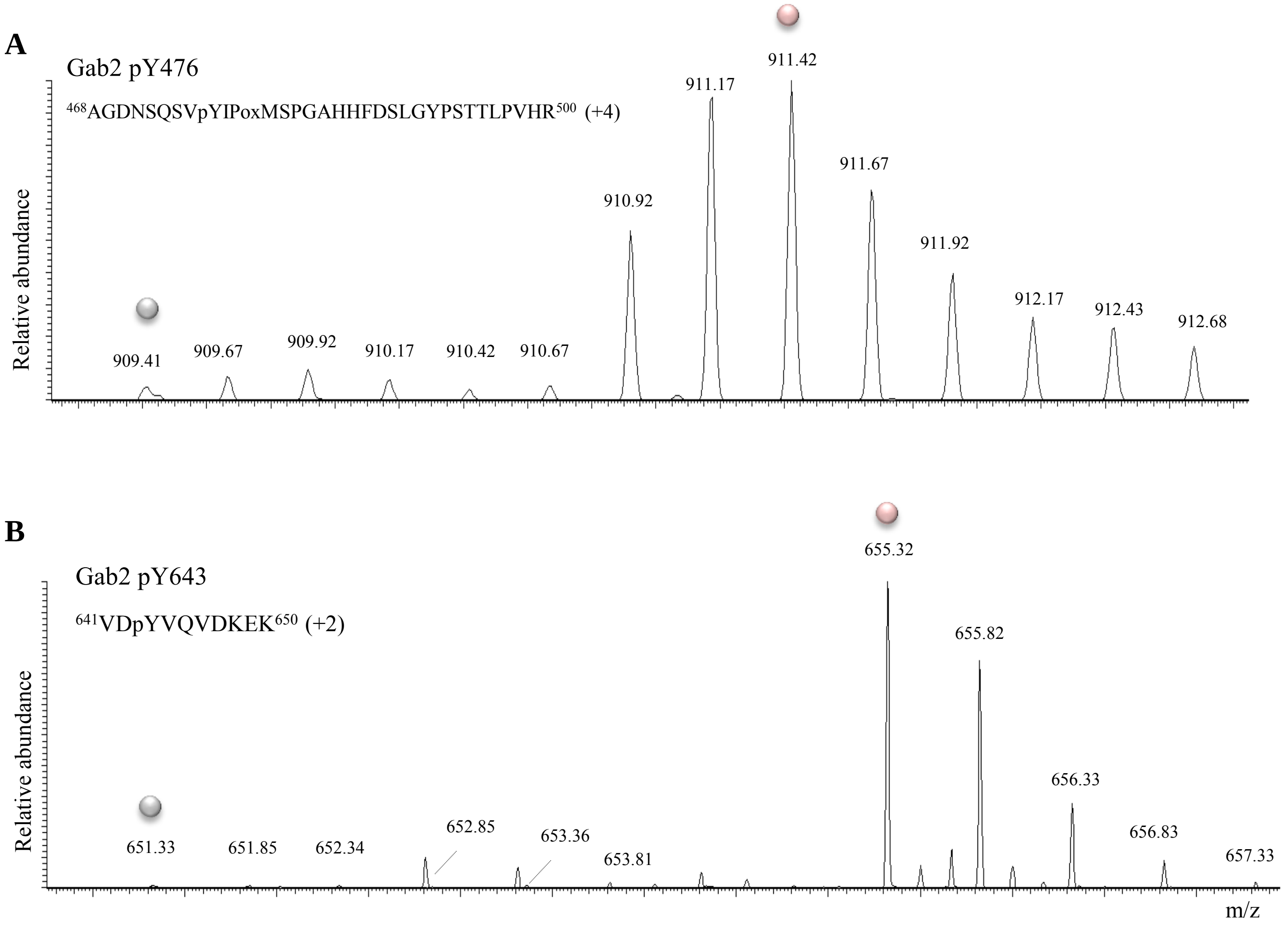

Supplementary Figure S2. MS spectra of pY-bearing peptides induced upon IL-2 stimulation. MS spectra of (*A*) pY468 and (*B*) pY643- containing phosphopeptides shows that both phosphopetides are more abundant in IL-2-treated condition (heavy SILAC condition, pink dot) in comparison to control condition (light SILAC condition, grey dot). Therefore, results indicate that GAB2 Y468 and Y643 become phosphorylated in IL-2-treated Kit225 T-cells in vivo.

Osinalde_Supplementary Figure S3

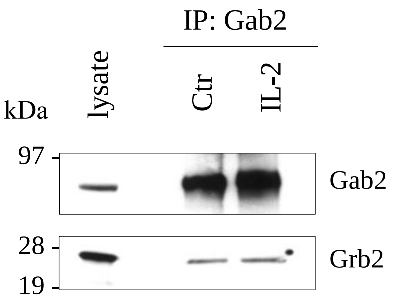

Supplementary Figure S3. Gab2 constitutively associates with Grb2 in Kit225 T-cells. Immunoprecipitation of Gab2 followed by immunobloting detection confirmed our MS-based data indicating that Gab2 and Grb2 constitutively interact in Kit225 T-lymphocytes

Supplementary Table S1. List of primers used in real time PCR analyses for mRNA expression

Gene	Forward primer (5´- 3´) Sequence	Reverse primer (5´- 3´) Sequence
Gab1	AAGACTACCTGTTGCTCATCAACTGT	GGACGTTATCATTGGAGTCTGTTTC
Gab2	GCCGGCACAATACAGAATTCA	GTGAGGCTGCCCTTGGTGT
Gab3	CACTGCAGCCCTGATGACTA	GAGAAAGCTGGTTCGACTG
β-actin	GACAGCAGTCGGTTGGAGCG	ACCTCCCCTGTGTGGACTTGG
